# Specular Microscopy of Human Corneas Stored in an Active Storage Machine

**DOI:** 10.3390/jcm11113000

**Published:** 2022-05-26

**Authors:** Thibaud Garcin, Emmanuel Crouzet, Chantal Perrache, Thierry Lepine, Philippe Gain, Gilles Thuret

**Affiliations:** 1 Ophthalmology Department, University Hospital, 42000 Saint-Etienne, France; philippe.gain@univ-st-etienne.fr (P.G.); gilles.thuret@univ-st-etienne.fr (G.T.); 2 Corneal Graft Biology, Engineering and Imaging Laboratory, BiiGC, EA2521, Federative Institute of Research in Sciences and Health Engineering, Faculty of Medicine, Jean Monnet University, Rue de la Marandière, 42000 Saint-Etienne, France; e.crouzet@univ-st-etienne.fr (E.C.); chantal.perrache@univ-st-etienne.fr (C.P.); thierry.lepine@institutoptique.fr (T.L.); 3 Laboratory Hubert Curien, Optics Institute Graduate School, 42000 Saint-Etienne, France; 4 Institut Universitaire de France, Boulevard Saint-Michel, 75005 Paris, France

**Keywords:** active storage machine, cornea, endothelium, endothelial cell density, eye bank, long-term storage, specular microscopy, viable endothelial cell density, image analysis

## Abstract

Purpose: Unlike corneas stored in cold storage (CS) which remain transparent and thin, corneas stored in organoculture (OC) cannot be assessed by specular microscopy (SM), because edema and posterior folds occur during storage and prevent from specular reflection. We previously developed an active storage machine (ASM) which restores the intraocular pressure while renewing the storage medium, thus preventing major stromal edema. Its transparent windows allow multimodal corneal imaging in a closed system. Aim: to present SM of corneas stored in this ASM. Methods: Ancillary study of two preclinical studies on corneas stored for one and three months in the ASM. A prototype non-contact SM was developed (CMOS camera, ×10 objective, collimated LED source, micrometric stage). Five non-overlapping fields (935 × 748 μm) were acquired in exactly the same areas at regular intervals. Image quality was graded according to defined categories (American Cornea Donor Study). The endothelial cell density (ECD) was measured with a center method. Finally, _SM_ECD was also compared to Hoechst-stained cell nuclei count (_Hoechst_ECD). Results: The 62 corneas remained thin during storage, allowing SM at all time points without corneal deconditioning. Image quality varied depending on donors and days of control but, overall, in the 1100 images, we observed 55% of excellent and 30% of good quality images. _SM_ECD did not differ from _Hoechst_ECD (*p* = 0.084). Conclusions: The ASM combines the advantages of CS (closed system) and OC (long-term storage). Specular microscopy is possible at any time in the ASM with a large field of view, making endothelial controls easy and safe.

## 1. Introduction

Short-term cold storage (CS) at 4 °C and long-term organ culture (OC) at 31–37 °C, the two storage methods in use worldwide, have a mandatory step of endothelial assessment. Endothelial cell density (ECD) remains the main quality criterion of a stored cornea because endothelial cells (EC), which ensure the stability of corneal transparency, do not renew in humans and because donor EC die faster during storage [1,2,3,4,5] and in recipients than do native, healthy EC [6].

To acquire images of the EC in these two storage systems, different methods exist. During CS, specular microscopy (SM) can provide non-invasive images of the EC through the wall of the corneal storage-viewing chamber or the vials, because the hyperosmolar medium keeps the cornea thin, and thus, its endothelial surface remains folded very little. Specular reflection is therefore possible on this nearly smooth surface. During OC, SM is impossible because the cornea quickly becomes edematous (its thickness can double), which induces numerous and deep posterior folds. To see the EC, it is therefore essential to use a transmitted light microscopy method (bright-field or phase-contrast) and to make the EC visible by temporarily dilating the intercellular spaces through a brief exposure to 0.9% sodium chloride, 1.8% sucrose, or hypotonic sodium-balanced salt. This endothelial control is therefore more restrictive and invasive. The two methods are therefore neither interchangeable nor equivalent.

To reproduce a more physiological corneal environment and break the vicious circle of hypotonia—corneal edema—deep posterior folds—endothelial lesion [2,3], we developed an active storage machine (ASM), also called a bioreactor, that reproduces a transcorneal pressure gradient equivalent to the intraocular pressure (IOP) and produces a renewal of the nutrient medium [7]. In two successive ex vivo experiments on pairs of fresh human corneas, we compared the ASM and OC using the same commercial storage medium for 1 month [8] and 3 months [9]. We demonstrated that, contrary to OC, the ASM prevented the corneas from majorly swelling, allowing them to be permanently ready for transplantation with significantly more viable EC than in OC. Furthermore, the better control of corneal thickness in the ASM allowed endothelial controls to be performed by SM.

In our ancillary study of two preclinical studies on corneas stored in the ASM for one and three months, we analyzed the quality of endothelial images obtained by SM.

## 2. Materials and Methods

### 2.1. Study Design: Ancillary Study of 1-Month and 3-Month Storage Experiments in the ASM

We analyzed all the SM images obtained on corneas stored in the ASM during the 2 validation studies of this new device [8,9]. All procedures conformed to the tenets of the Declaration of Helsinki for biomedical research involving human subjects. The French Agence de la Biomédecine specifically authorized the retrieval of corneas for these preclinical studies (PFS15-008 and PFS16-010). For the fifty corneas stored in the ASM for one month, EC counts were performed on day (D) 2, 26, and 28. For the twelve corneas stored in the ASM for three months, EC counts were performed on D2, 23, 44, 65, 86, and 88. The complete system of the ASM, control panels excepted, was placed in a 31 °C dry 5% CO_2_ incubator. The medium used was CorneaMax, a CE marked OC Dulbecco’s Modified Eagle Medium–based medium containing 2% FCS, penicillin, and streptomycin (Eurobio, les Ulis, France). The medium flow rate was set at 2.6 μL/min (normal aqueous humor flow rate) and the transcorneal pressure gradient at 21.5 mmHg (upper limit of normal IOP in humans) with atmospheric pressure as a reference.

In these two studies, we performed a measurement of viable ECD (vECD) by a destructive technique known as triple Hoechst-Ethidium-Calcein-AM staining, which we had previously described, at the end of storage [10,11] (*detailed in part 2.5*). We therefore used the images of the Hoechst staining of all nuclei to obtain a very accurate _Hoechst_ECD measurement of very large areas. We then compared the SM endothelial counts (_SM_ECD) with the _Hoechst_ECD, which was considered our gold standard.

### 2.2. Building of a Customized Specular Microscope

A custom SM was built because the 3 commercial eye bank SMs (ebSM) available in our laboratory were not adapted for the ASM. The standard equipment of the HAI EB-2000xyz (HAI, Lexington, KY, USA) and of the EB-10 and Cell-ChekD(+) (KONAN, Nishinomiya, Japan) could not accept the ASM cassette on their stage and/or the working distance of their objective was too short.

**Figure 1:**
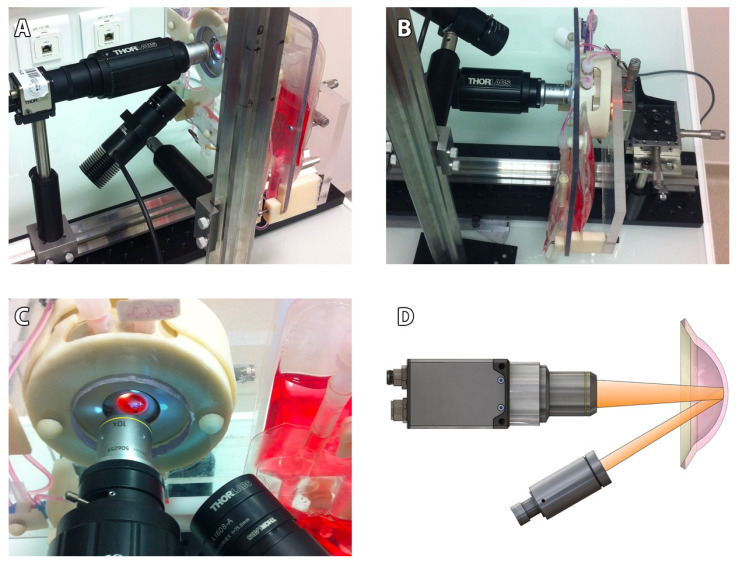
**Custom-made specular microscope prototype adapted for our active storage machine (ASM).** (**A**) General view of the optical bench, the camera and its objective, the led source, and the 3D-printed support intended to receive the ASM. Note the working distance, the collimated source, and the diaphragm produce a light beam focused on endothelial side of the cornea. (**B**) The ASM could be moved precisely thanks to the micrometric translation XYZ stage. (**C**) Close-up view, with one quadrant. (**D**) Schematic view of specular reflection from endothelial side of the cornea. The collimated source illuminated a corneal area, and the camera recorded the specular reflection.

Our non-contact SM comprised (Figure 1): an optical bench (Thorlabs, Newton, MA, USA), a CMOS camera (DCC3240M, Thorlabs) equipped with a long working distance (10×/0.25, N Plan,∞/−/B) objective (Leica, Wetzlar, Germany) and driven by Scientific Imaging Thorcam v2.6.7064 software, an LED driver on constant current mode (LEDD1B, Thorlabs), an external LED source (MCWHL5-C2, Thorlabs) mounted and collimated with a diaphragm (SM1D12, Thorlabs) and a plano-convex lens (f30mm LA1805-A, Thorlabs), and a certified micrometric translation XYZ stage (M-UMR5.16A; sensitivity 0.1 µm for the Z axis (SM-13); 1 µm for the XY axis (BM11.10), Newport, Irvine, CA, USA) activated manually and connected to a 3D-printed support intended to receive the ASM. Accuracy in XYZ was verified using calibrated ceramic gage blocks (Mitotoyo, Roissy, France). The resulting field of view of our SM was 935 × 748 μm, with 1280 × 1024 pixels TIFF images’ acquisition (2.6Mo). Image calibration was verified using a 10 mm scale with 50 µm divisions (R1L3S1P, Thorlabs).

**Figure 2:**
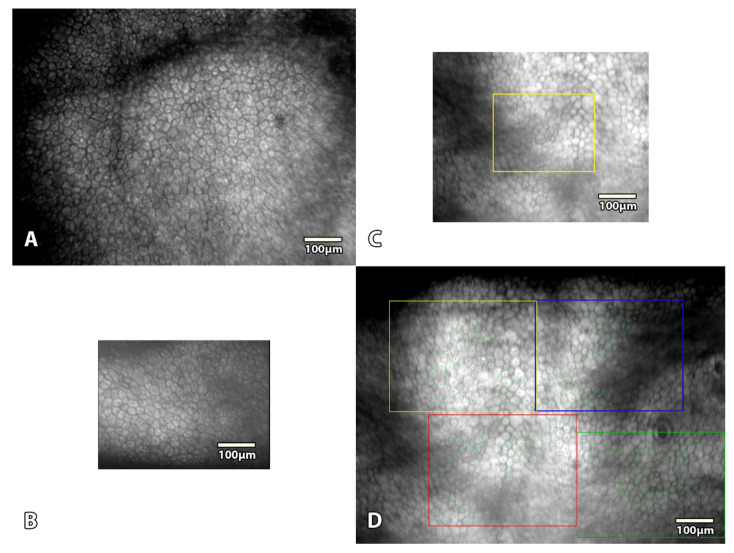
**Comparison between our prototype and 3 eye banks’ specular microscopes (SM).** (**A**) The custom-made prototype SM with 935 × 748 μm and 1280 × 1024 pixels TIFF image. The entire surface could be analyzed. (**B**) The HAI EB-2000xyz had a 368 × 490 µm field of view (480 × 640 pixels BMP image) and analysis (3.9 times smaller). (**C**) The KONAN EB-10 had a 480 × 600 µm field of view (595 × 794 pixels BMP image) and a 280 × 200 µm field of analysis (respectively 2.4 and 12.5 times smaller). (**D**) The KONAN Cell-ChekD(+) had a 1000 × 750 µm field of view (1296 × 972 pixels BMP image) and up to 4 areas of 400 × 300 µm field of analysis (respectively 1.1 times larger but 1.5 times smaller).

Although no direct comparison was possible between the 3 commercial microscopes and our prototype, we used an additional cornea stored at 4 °C in Optisol-GS (Bausch & Lomb, Laval, QC, Canada) in a corneal storage viewing chamber (Krolman, Boston, MA, USA) to indirectly compare the surface of the observation fields (Figure 2).

### 2.3. Image Acquisition

Endothelial images were acquired through the endothelial glass window (flat sapphire glass, diameter 23.7 mm, thickness 0.9 mm, France Fourniture Horlogerie, Vence, France) of the ASM without deconditioning the corneas. Both ends of the endothelial chamber tubes were temporarily clamped to maintain pressure inside the endothelial chamber and allow the ASM’s removal from the CO_2_ incubator (in this experimental version of the ASM, tubes were permeable to gas, and the OC medium used a bicarbonate buffer, making it mandatory to use a 5% CO_2_ atmosphere to maintain a physiologic pH).

**Figure 3:**
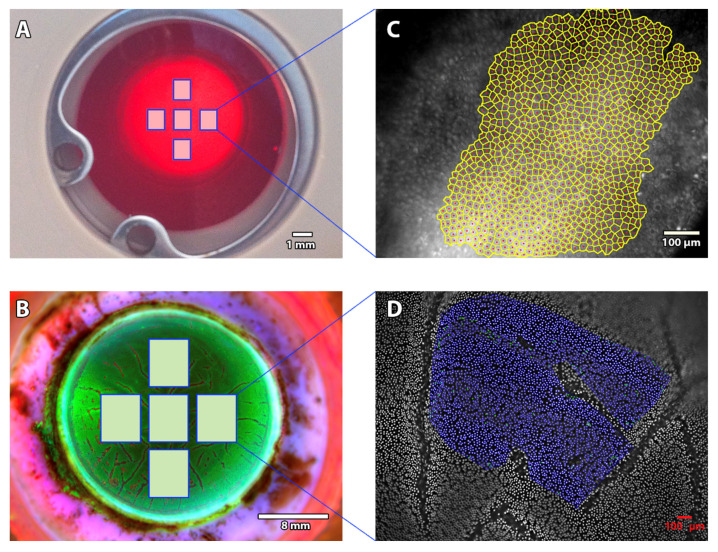
**Image acquisition methods.** (**A**) Five non-overlapping fields were captured with our non-contact specular microscope. (**B**) Pancorneal triple Hoechst-Etidium-Calcein staining. Five non-overlapping fields were analyzed. In the present study, we used the Hoechst staining image (100% visible nuclei) as a gold standard to analyze the accuracy of specular count. (**C**) Each field was analyzed with validated variable-frame center method [12,13]. (**D**) A close-up view of one of the fields, nuclei of the endothelial cells were counted thanks to the CorneaJ plugin [10,11].

We standardized the image acquisition: To retrieve specular reflection, the LED source was placed on the endothelial side of the cornea at 31 ± 4° from the camera optical axis. To modulate the contrasts and observe either the EC or the epithelium, the LED source was also specifically inclined on the Y axis (15 ± 4°). The camera software settings were presettled to optimize image quality: pixel clock (20 MHz), fixed frame rate (4.20FPS), fixed exposure time (16.70 ms), pixel data format 8-bit monochrome, gamma software (1.00), automatic image gain, and black level offset auto-adaptation according to each LED source position. Five non-overlapping fields of EC—one in the center and one per quadrant—were acquired inside the 8 central mm (the area usually grafted) (Figure 3). For each cornea, exactly the same area of each field was manually acquired, thanks to our calibrated device, by certified micrometers in this prototype version (position recorded for each cornea), at different periods for the 1-month [8] and 3-month [9] storage periods. Image acquisition took fewer than 10 min.

### 2.4. Image Quality Classification

Endothelial SM images were classified in reference to the Specular Microscopy Ancillary Study (SMAS) of the American Cornea Donor Study [14,15]. We categorized images as either analyzable (subclassified as excellent, good, and fair) or unanalyzable. Briefly, excellent images had at least 50 and as many as 150 cells contiguous to each other that could be counted, with all distinct cell borders, boundaries, and centers across a single image. Good images had at least 50 and as many as 150 cells from variable frames encompassing a minimum of 15 cells contiguous to each other for each variable frame, with sufficient distinct cell borders, boundaries, and centers across a single image. Fair images had at least 50 cells from variable frames encompassing a minimum of 15 cells contiguous to each other for each variable frame, with up to 25% indistinct borders, boundaries, and centers of cells across a single image. Unanalyzable images had an uncountable ECD: fewer than 50 cells with distinct borders, boundaries, and centers from a single image of the endothelium could be counted from variable frames encompassing a minimum of 15 cells contiguous to each other for each variable frame.

### 2.5. Endothelial Cell Count Methods

To determine the ECD, cells were counted on each field, and the mean of the five was calculated. We used the ImageJ freeware (https://fiji.sc (accessed on 1 November 2016)) with a customized plugin ECD3D [16]. The observer chose areas where EC were most clearly visible and avoided folds when present. The reconstructed full-field image ECD was determined using a validated variable-frame center method [12,13]. Briefly, the center of all EC constituting a continuous group was pointed manually, and the group’s boundaries were drawn manually. Cell borders were then automatically reconstructed using Vonoroi segmentation and carefully verified by one skilled observer (TG), who made all necessary corrections. The process was similar to that used in numerous eye banks except for the number of EC manually pointed, which in this study was dramatically elevated (at least 200 EC per field captured–4 times more than in SMAS—and as many as possible in the greatest cell area) to increase cell count reliability [14,15]. The mean ± standard deviation (SD) of the ECD determined in the five fields was calculated for each cornea in the ASM at different time points.

In addition, a final measurement was performed at the end of storage (D28 or D88) in order to determine the vECD by using a triple HEC staining with the CorneaJ plugin for image analysis, as previously reported [10,11]. Pancorneal viability was measured thanks to the mean of five non-overlapping fields of EC acquired inside the 8 central mm (the area usually grafted) by one skilled observer (TG). To assess the accuracy of ECD counted by SM (_SM_ECD), we compared it with ECD measured by counting Hoechst-stained cell nuclei in large variable frames (_Hoechst_ECD) (hereafter referred to as the “histology count”) (Figure 3). All specular and Hoechst counts were blinded. They were compared later.

### 2.6. Statistics

The normality of continuous data distribution was analyzed with the Shapiro-Wilk test, with a non-normality threshold set at 5%. Normally distributed data were described by their mean ± SD. Continuous abnormally distributed variables were summarized as median (10–90 percentiles). The non-parametric Wilcoxon signed-rank test was used when the variable followed an abnormal distribution, and a *t*-test was used when the variable followed a normal distribution. All tests were two-tailed and paired. Rejection of the null hypothesis was defined as α < 0.05. The Holm–Sidak method was used when multiple comparisons occurred (ANOVA). Statistical analyses were performed using SPSS 25.0 (IBM Corp, Armonk, NY, USA).

## 3. Results

### 3.1. Baseline Donor Characteristics

Overall, 62 corneas were analyzed: 50 and 12 from one- and three-months’ storage, respectively. Donors were 29 females and 35 males, with a mean age of 79 ± 12 years (range 49–97). Mean time from death to procurement was 16 ± 5 h (range 4.30–24). Of the total, 32 eyes (26%) had undergone cataract surgery (same proportion in both groups, ASM and OC).

### 3.2. Image Quality Classification

**Figure 4:**
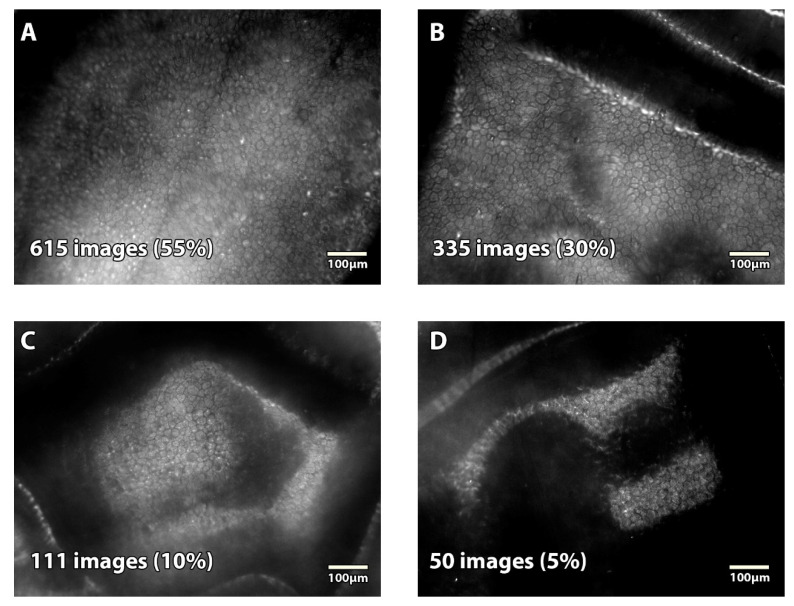
**Representative image quality obtained with the prototype specular microscope on corneas stored in the active storage machine**. Grading was done using criteria of the Specular Microscopy Ancillary Study [14,15]. (**A**) Excellent-quality image with an ECD of 3022 cell/mm^2^. (**B**) Good-quality image with an ECD of 2637 cell/mm^2^. (**C**) Fair-quality image with an ECD of 2689 cell/mm^2^. (**D**) Unanalyzable image.

Of the 1110 images, 1060 (95%) were analyzable—615 (55%) were excellent, 335 (30%) good, 110 (10%) fair—and 50 (5%) unanalyzable (Figure 4). Seven hundred and fifty images were analyzed for the 1-month study and 360 for the 3-month study. The percentages of the different image quality groups (excellent, good, fair, and unanalyzable) did not differ significantly between the two studies (*p* = 0.240): 59%, 28%, 9%, 4% for the 1-month study; and 47%, 36%, 11%, 6% for the 3-month study. Table 1 shows the details of image quality classification for both studies.

### 3.3. Number of Cells Counted per Image

**Figure 5:**
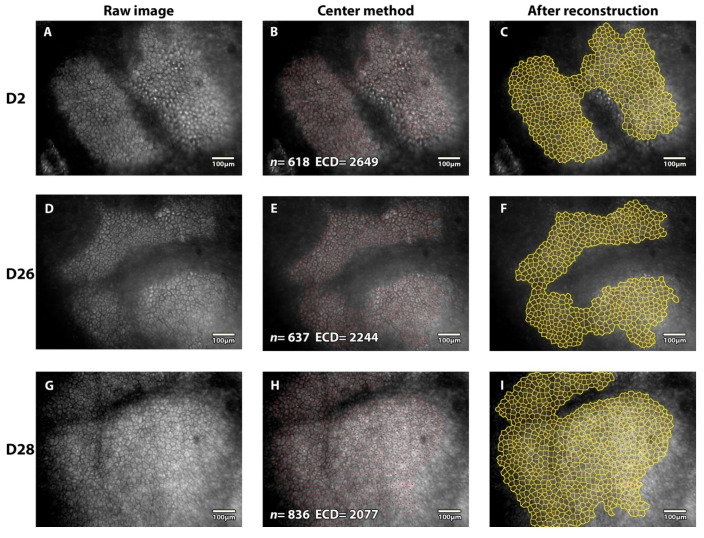
**Example of specular microscopy images of the same area of the same cornea stored in the active storage machine for one month**. An accurate monitoring of endothelial cell density was possible at day (D) 2, 26, and 28. *n* = number of cells counted; ECD endothelial cell density in cells/mm^2^. At D2, (**A**) raw image with endothelial cells, (**B**) each center was pointed according to the center method, (**C**) cells borders and each center were marked thanks to ImageJ plugin ECD3D [16]. At D26, (**D**) comparative raw image showed larger cells, (**E**) ECD count was determined with the same method, (**F**) reconstruction allowed global view of the studied sample. At D28, (**G**) progressive redistribution of cells occurred, (**H**) more cells were counted on a larger area inside the same field of view (less endothelial folds), (**I**) reconstruction highlighted polymegethism and pleiomorphism.

The mean number of EC counted per image was 1360 ± 433 (range 224–3022), depending on the ECD. In both the 1- and the 3-month studies, the number of counted cells per field/image increased over time (ANOVA, *p* < 0.001). Figure 5 shows an example of the follow-up of the same area at different times during one month. Details for each study are provided in the Supplemental Data (Table S1, Figure S1, Video S1).

### 3.4. Comparison of the SM Counts with the Histology Count

Overall, at the end of storage, _SM_ECD did not significantly differ from _Hoechst_ECD, with 2209 ± 363 versus (vs.) 2251 ± 414 cells/mm^2^, respectively (*p* = 0.084). For the 1-month storage study, ECD were 2299 ± 332 (_SM_ECD) vs. 2344 ± 392 cells/mm^2^ (_Hoechst_ECD) (*p* = 0.138). For the 3-month storage study, ECD were 1831 ± 213 (_SM_ECD) vs. 1863 ± 248 cells/mm^2^ (_Hoechst_ECD) (*p* = 0.081).

### 3.5. Others Options of the Prototype Specular Microscope

**Figure 6:**
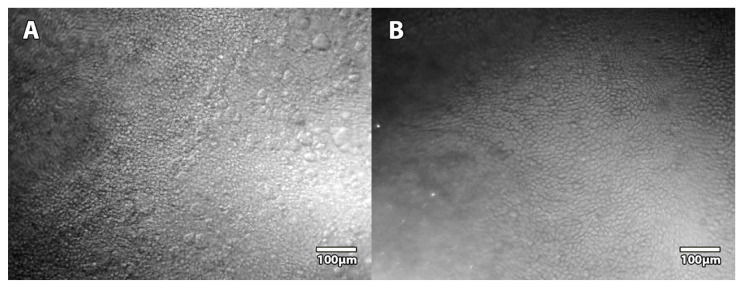
**Specular microscopy images of the epithelium of corneas stored in the active storage machine.** Thanks to the precision of the Z axis of the microscope stage and the variation of angulation of the LED source, we were able to record epithelium images at different depths: superficial layers (**A**) and more basal layers (**B**).

The prototype non-contact SM allowed movie recording in the same area by modulating depth on the Z axis to explore the greatest area of endothelium and to choose the most representative area for each field (Video S2). In addition, epithelial cells could also be observed with the same field of view as the endothelial imaging (Figure 6).

## 4. Discussion

The ASM is the first device for corneal graft storage that restores transcorneal pressure gradient equivalent to the IOP while renewing the storage medium. We reported better endothelial survival in the ASM versus the OC and demonstrated its superiority over OC in terms of the preservation of endothelial survival [8,9]. These two initial studies allowed us to transfer the intellectual property to a company that aims to industrialize the ASM so that all the eye banks that wish to use it can do so in the near future. Since the ASM was designed as a totally closed system, it would be inappropriate to have to extract the cornea from the ASM to perform the endothelial controls. Because IOP is involved in the control of corneal hydration ex vivo, the ASM limits the development of stromal edema during long-term storage in a conventional OC medium. In this study carried out before the transfer of the ASM to an industrial company and fully independently of the industrial company, we showed that the endothelium and the epithelium of long-term-stored corneas in the ASM can be observed by SM. To evaluate the quality of the SM images, we used all 1100 images—almost two times as many images as in SMAS [14]—from the two ASM validation studies (1 and 3 months), acquired and analyzed with a standardized method. We decreased sampling fluctuations with at least 200 EC counted per image, meaning 1000 EC counted per cornea at different time points. Our SM counts were reliable: the _SM_ECD of the last count performed at the end of storage was comparable to the histological count.

The association of the ASM and our prototype SM that we developed has several advantages over existing systems, particularly: (1) The field of view is wider, and the image can be analyzed on its entire surface; (2) The sealed cornea in the ASM and the micrometric stage allow it to have exactly the same position as the ASM along the storage. If the observer wishes, it is thus possible to acquire and analyze, for each cornea, exactly the same endothelial area at different times and to calculate a cell loss rate precisely; (3) With the ASM, it is not necessary to warm up the cornea to see the EC. We showed that SM was possible at any time. When stored at 4 °C, it is essential to warm up the cornea for several hours to acquire satisfactory SM images [17]. It is likely that, at 4 °C, the EC themselves exhibit edema that prevents imaging of cell borders, while the EC are in a more physiological status in the ASM.

During the endothelial controls of corneas in OC, many European eye banks use trypan blue staining to highlight dead EC. However, this counting is not standardized: the staining of the nuclei is often very low, and the count methods do not allow the counting of all the stained cells on a representative surface. The percentage of dead EC is therefore very imprecise. In practice, trypan blue is mainly used to identify large areas of dead cells that may indicate trauma or herpetic infection. Vital staining is impossible in a closed system; thus, it is advisable to repeat EC controls by SM just before corneal graft delivery. Endothelial necrosis at 31–34 °C destroys the endothelium within a few days by cytopathic effect and progression by contiguity, and it will be detected by SM where no EC is visible.

We used the image-quality classification system defined in the SMAS of the CDS, which was referenced in terms of SM image analysis [14]. Interestingly, this prospective study analyzed 688 endothelial images of corneas stored in Optisol-GS, in corneal storage-viewing chambers, submitted by 23 eye banks, acquired with 5 different SM (BioOptics Inc. (Portland, OR, USA); CooperVision (no longer manufactured); HAI Laboratories, Inc. (Lexington, MA, USA); Konan Inc. (Phoenix, AZ, USA); or Tomey (Phoenix, AZ, USA)), and analyzed by a central reading center. They obtained 663 (96%) analyzable images (versus 95% in the present study). However, their image quality seemed inferior, with 6% excellent, 44% good, and 47% fair (versus 55%, 30%, and 10% respectively in our study).

There are limitations to our study. Our experimental prototype SM is not CE-marked or FDA-approved and does not include dedicated cell count software. Conversely, the commercial ebSM are not compatible with the ASM without modifications. It should be possible to adapt the ebSM, requiring FDA or CE approbation: the stages must be modified to receive the ASM cassette, which is much bigger than a conventional corneal storage-viewing chamber; in some cases, the objective must be to adapt its working distance and likely the lighting mode, taking into account the distances between the ASM window and the corneal endothelium. Finally, despite our standardized image acquisition, the settings required rigorous experience to acquire and count images. An all-in-one, more automated commercial version should be developed for routine use in eye banks.

## 5. Conclusions

Thanks to the control of corneal hydration and the reduction of endothelial folding, corneal grafts stored long-term in our ASM can benefit from endothelial controls with SM at any time, with an image quality comparable to or better than short-term cold-stored corneas.

## Figures and Tables

**Table 1:** Image quality classification obtained with specular microscopy of corneas stored in the active storage machine.

	1-Month Study [8]			3-Month Study [9]								
	D2	D26	D28	D2	D23	D44	D65	D86	D88	1 Month *n* (%)	3 Months *n* (%)	Total *n* (%)
*n* Excellent	146	150	149	26	33	32	30	25	24	445 (59)	170 (47)	615 (55)
*n* Good	65	70	70	19	20	21	24	23	23	205 (28)	130 (36)	335 (30)
*n* Fair	27	22	21	10	5	4	4	8	9	70 (9)	40 (11)	110 (10)
*n* Unanalyzable	12	8	10	5	2	3	2	4	4	30 (4)	20 (6)	50 (5)
TOTAL	250	250	250	60	60	60	60	60	60	750	360	1110

*n* = number of images analyzed per category; D = day.

## Data Availability

The datasets used and/or analyzed during the current study are available from T.G. or G.T. on reasonable request.

## Supplementary Materials

The following supporting information can be downloaded at: https://www.mdpi.com/article/10.3390/jcm11113000/s1, Table S1: Number of cells counted per image in the 1- and 3-month studies; Figure S1: Time lapse of the same endothelial area during three months in the active storage machine. The same area for each field was precisely acquired repeatedly from Day 2 to Day 86, thanks to the micrometric stage of our specular microscope. Green arrow indicates the redistribution of endothelial cells at the same point of the field. Endothelial cell density decreased over time with larger cells and a progressive slight increase in polymegethism and pleiomorphism; Video S1: Time lapse showing EC redistribution over three months; Video S2: Movie recording example on a defined area.
