# Supplementary material for: Specular Microscopy of Human Corneas Stored in an Active Storage Machine
Source: J Clin Med. 2022 May 26;11(11):3000. doi: 10.3390/jcm11113000 (PMC9181187; doi:10.3390/jcm11113000)
Supplement: Supplementary file 1 [file jcm-11-03000-s001.zip › jcm-1693536-supplementary files.pdf]

Journal of
Clinical Medicine

MDPI

Article

Specular Microscopy of Human Corneas Stored in an Active Storage Machine

Thibaud Garcin [1,2,*], Emmanuel Crouzet [2], Chantal Perrache [2], Thierry Lepine [2,3], Philippe Gain [1,2] and Gilles Thuret [1,2,4]

[1] Ophthalmology Department, University Hospital, 42000 Saint-Etienne, France; philippe.gain@univ-st-etienne.fr (P.G.); gilles.thuret@univ-st-etienne.fr (G.T.)
[2] Corneal Graft Biology, Engineering and Imaging Laboratory, BiiGC, EA2521, Federative Institute of Research in Sciences and Health Engineering, Faculty of Medicine, Jean Monnet University, Rue de la Marandière, 42000 Saint-Etienne, France; e.crouzet@univ-st-etienne.fr (E.C.); chantal.perrache@univ-st-etienne.fr (C.P.); thierry.lepine@institutoptique.fr (T.L.).
[3] Laboratory Hubert Curien, Optics Institute Graduate School, 42000 Saint-Etienne, France
[4] Institut Universitaire de France, Boulevard Saint-Michel, 75005 Paris, France
* Correspondence: t.garcin@univ-st-etienne.fr; Tel.: +33-(0)4-77-12-77-93; Fax: +33-(0)4-77-12-09-95

Citation: Garcin, T.; Crouzet, E.; Perrache, C.; Lepine, T.; Gain, P.; Thuret, G. Specular Microscopy of Human Corneas Stored in an Active Storage Machine. *J. Clin. Med.* **2022**, *11*, 3000. https://doi.org/10.3390/jcm11113000

Academic Editors: Vito Romano, Yalin Zheng and Mariantonia Ferrara

Received: 6 April 2022
Accepted: 24 May 2022
Published: 26 May 2022

Publisher's Note: MDPI stays neutral with regard to jurisdictional claims in published maps and institutional affiliations.

Copyright: © 2022 by the authors. Licensee MDPI, Basel, Switzerland. This article is an open access article distributed under the terms and conditions of the Creative Commons Attribution (CC BY) license (https://creativecommons.org/licenses/by/4.0/).

SUPPLEMENTARY DATA

Number of cells counted per image (Table S1)

For the 1-month storage study, the overall mean number of counted cells per SM image was 1364±510 (range 224-3022) at each time point. The mean number of counted cells per SM image increased over time (ANOVA, P<0.001): at D2 1008±426 cells (range 224-3022), at D26 1437±415 cells (range 813-2630), at D28 1646±470 cells (range 949-2866). The final endothelial assessment with $_{\text{Hoescht}}$ECD allowed extra-large wide field of analysis with a mean of 4768±1769 nuclei cells (range 2751-9122) analyzed per image.

For the 3-month storage study, the overall mean number of counted cells per SM image was 1353±194 (range 877-1729) at each time point. The mean number of counted cells per SM image increased similarly over time (ANOVA, P<0.001): at D2 1033±105 cells (range 877-1286), at D23 1241±95 cells (range 1085-1456), at D44 1360±81 cells (range 1282-1597), at D65 1433±88 cells (range 1284-1583), at D86 1493±68 cells (range 1355-1657), and at D88 1558±67 cells (range 1475-1729). The final endothelial assessment $_{\text{Hoescht}}$ECD allowed extra-large wide field of analysis with a mean of 3235±266 nuclei cells (range 2954-4035) analyzed per image.

Table S1. Number of cells counted per image in the 1 and 3-month studies.

**1-month Study [8]**	**D2**	**D26**	**D28**	**Final Hoechst**		
Mean N of cells±SD	1008±426	1437±415	1646±470	4768±1769		
**3-month Study [9]**	**D2**	**D23**	**D44**	**D65**	**D86**	**D88**	**Final Hoechst**
Mean N of cells±SD	1033±105	1241±95	1360±81	1433±88	1493±68	1558±67	3235±266

N = number analyzed ; D = day ; SD = standard deviation

Thanks to our custom SM, a precise of follow-up of the same area all along the storage was possible. **Figure S1** and **Video S1** highlight EC redistribution over three months.

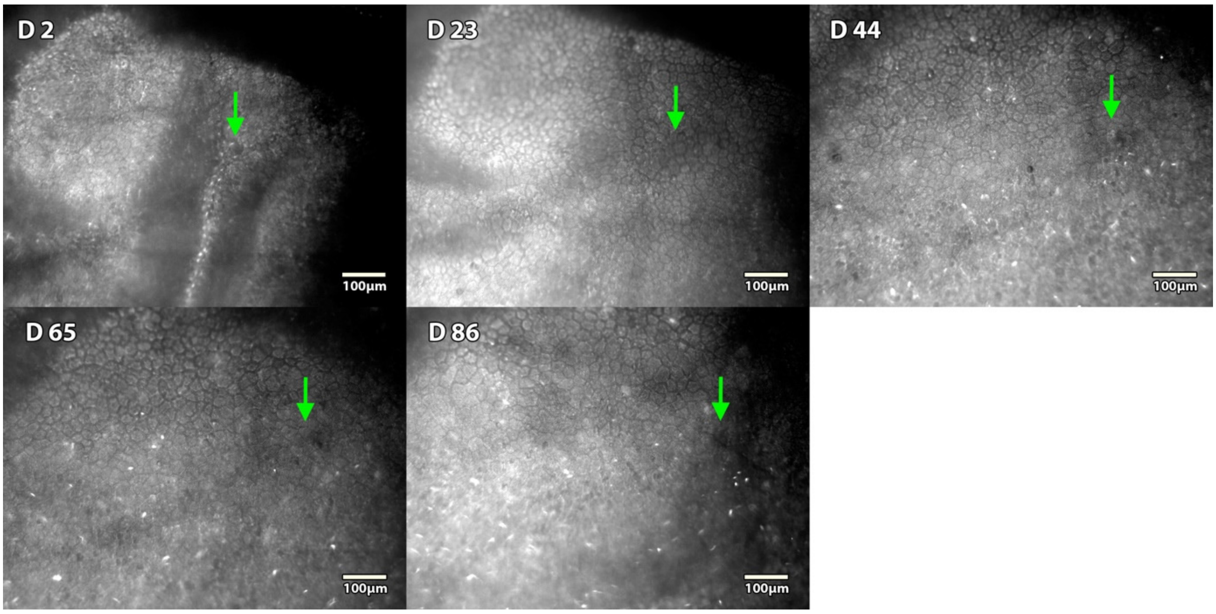

Figure S1. Time lapse of the same endothelial area followed during three months in the active storage machine. The same area for each field was precisely acquired repeatedly from Day 2 to Day 86 thanks to micrometric stage of our specular microscope. Green arrow indicated the redistribution of endothelial cells at the same point of the field. Endothelial cell density decreased over time, with larger cells, a progressive slight increased polymegethism and pleiomorphism.

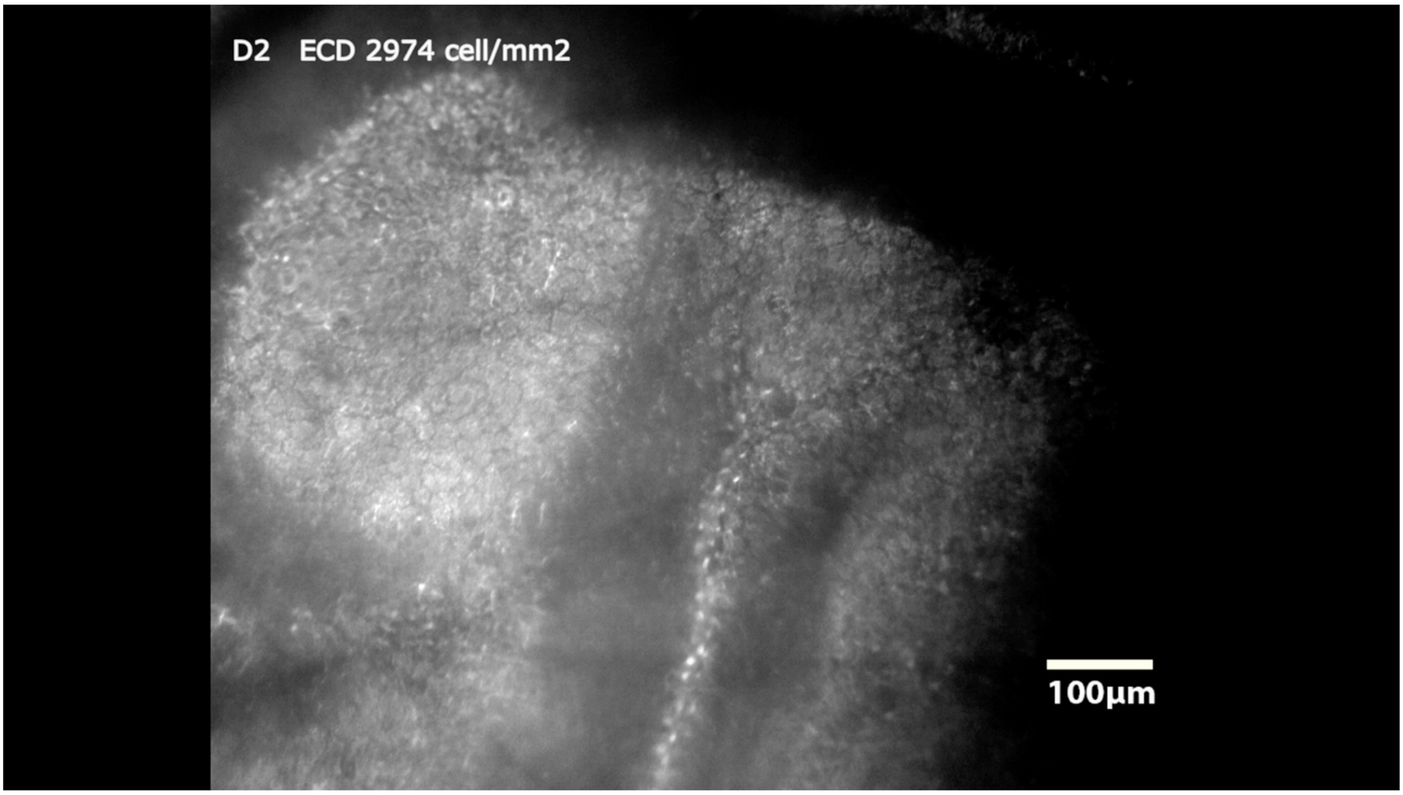

Video S1. Time lapse showing EC redistribution over three months.

Our custom allowed movie recording in the same area, by modulating depth on Z axis (**Video S2**).

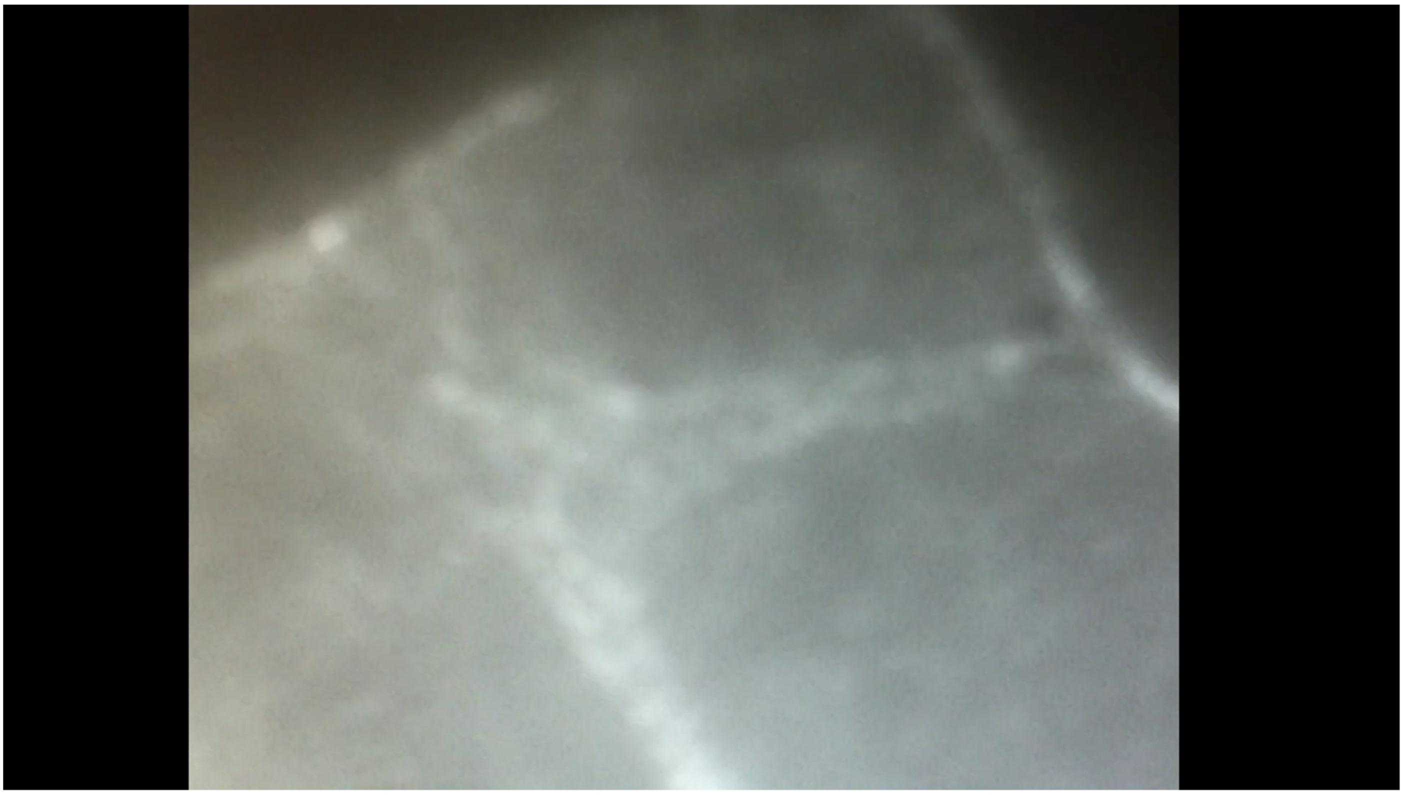

Video S2. Movie recording example on a defined area.
